# The Origins and Functions of Tissue-Resident Macrophages in Kidney Development

**DOI:** 10.3389/fphys.2017.00837

**Published:** 2017-10-25

**Authors:** David A. D. Munro, Jeremy Hughes

**Affiliations:** ^1^Centre for Integrative Physiology, School of Biomedical Sciences, University of Edinburgh, Edinburgh, United Kingdom; ^2^MRC Centre for Inflammation Research, Queens Medical Research Institute, University of Edinburgh, Edinburgh, United Kingdom

**Keywords:** metanephros, renal, phagocyte, monocyte, ontogeny, branching morphogenesis, angiogenesis, nephron

## Abstract

The adult kidney hosts tissue-resident macrophages that can cause, prevent, and/or repair renal damage. Most of these macrophages derive from embryonic progenitors that colonize the kidney during its development and proliferate *in situ* throughout adulthood. Although the precise origins of kidney macrophages remain controversial, recent studies have revealed that embryonic macrophage progenitors initially migrate from the yolk sac, and later from the fetal liver, into the developing kidney. Once in the kidney, tissue-specific transcriptional regulators specify macrophage progenitors into dedicated kidney macrophages. Studies suggest that kidney macrophages facilitate many processes during renal organogenesis, such as branching morphogenesis and the clearance of cellular debris; however, little is known about how the origins and specification of kidney macrophages dictate their function. Here, we review significant new findings about the origins, specification, and developmental functions of kidney macrophages.

## Introduction

Macrophages are specialized (“professional”) phagocytic cells that facilitate wide-ranging processes in diverse species. As well as their roles in host immunity and inflammation, macrophages are important in processes such as limb regeneration in salamanders (Godwin et al., 2013), stripe formation and blood vessel repair in zebrafish (Liu et al., 2016; Eom and Parichy, 2017), and synaptic pruning during brain development in the mouse (Paolicelli et al., 2011; Zhan et al., 2014).

Macrophages were first described in detail by Ilya Metchnikoff, who discovered their ability to engulf, digest, and destroy cellular components from living and dead microbial and host cells (Metchnikoff, 1905; Gordon, 2008). In 1924, the term “reticuloendothelial system” was coined to describe the system of phagocytic cells and their antecedents (based on the observation that phagocytes often form reticular networks around endothelia; Aschoff, 1924; Yona and Gordon, 2015). Subsequently, in 1969, prominent immunologists decided that the term “reticuloendothelial” was no longer adequate to describe this system; it was therefore relabeled as “the mononuclear phagocyte system” to reflect increased knowledge about the functions and morphology of monocytes, dendritic cells, and macrophages, and the derivation of these cells from the bone marrow (van Furth et al., 1972; Yona and Gordon, 2015). More recently, the notion that phagocytic cells derive chiefly from adult bone marrow-derived monocytes has been challenged, as evidence has accumulated showing that most adult tissue-resident macrophages derive from embryonic macrophages (Schulz et al., 2012; Hashimoto et al., 2013; Epelman et al., 2014a; Hoeffel et al., 2015; Sheng et al., 2015).

The first data highlighting that macrophage precursors exist in the yolk sac and fetal liver of the early embryo were published over 40 years ago (Moore and Metcalf, 1970; Cline and Moore, 1972). We now have a detailed understanding of how these cells contribute to various adult tissue-resident macrophage populations, and there are many informative reviews on this subject (Epelman et al., 2014b; Hoeffel and Ginhoux, 2015; Varol et al., 2015; Ginhoux et al., 2016). However, no reviews have focused specifically on describing the origins of kidney macrophages. Moreover, limited information is available about the functions of macrophages within the developing kidney. Here, we provide a comprehensive overview of the available data regarding the origins, specification, and functions of kidney macrophages in renal development. In this review, we also relate recent findings to emerging concepts in the field of macrophage research and highlight important questions that are still to be addressed.

## Origins of tissue-resident macrophages

During embryogenesis, macrophages colonize developing organs in overlapping waves (Schulz et al., 2012; Hoeffel et al., 2015; Sheng et al., 2015). Due to their remarkable capacity to self-renew *in situ*, many of these embryonic macrophages remain in adult tissues (Merad et al., 2002; Ajami et al., 2007; Hashimoto et al., 2013; Sieweke and Allen, 2013; Hoeffel et al., 2015). This fact contradicts the long-held belief that adult tissue-resident macrophages derive from, and are continually replenished by, circulating monocytes (van Furth and Cohn, 1968; van Furth et al., 1972). In this review, we describe tissue-resident macrophage origins in the mouse, as there is a paucity of information about their origins in the human.

The yolk sac provides the first wave of macrophages during development, commencing at embryonic day 7 (E7), before the embryonic circulation is established (Moore and Metcalf, 1970; Palis et al., 1999; McGrath et al., 2003). Erythro-myeloid progenitors (EMPs) emerge from the blood islands and capillary endothelia of the yolk sac (Kasaai et al., 2017). These cells form independently of c-Myb, a master transcriptional regulator of hematopoiesis (Sumner et al., 2000; Sandberg et al., 2005; Gomez Perdiguero and Geissmann, 2013). Rather than passing through an intermediate monocytic phase, c-Myb-independent EMPs directly acquire a core macrophage transcriptional programme and differentiate into pre-macrophages (pMacs) before maturing into tissue-resident macrophages (Takahashi et al., 1989; Schulz et al., 2012; Mass et al., 2016). At E8.5, when the yolk sac vasculature connects with the embryonic vasculature, these yolk sac macrophages migrate throughout the embryo and enter tissues such as the early brain and liver (Kierdorf et al., 2013; Gomez Perdiguero et al., 2015).

From mouse E8.5, a second set of EMPs, which are c-Myb-dependent, emerge from hemogenic endothelia in the yolk sac (Hoeffel et al., 2015). Many of these EMPs travel through the embryonic vasculature to colonize the fetal liver (McGrath et al., 2015; Mass et al., 2016). Due to their rapid expansion in the liver, by E11.5, the number of liver-EMPs exceeds the number of yolk sac-EMPs by 25-fold (Gomez Perdiguero et al., 2015). Concurrent with their expansion in the liver, the c-Myb-dependent EMPs differentiate into monocytic intermediates (Hoeffel et al., 2015) and/or into pMacs (Mass et al., 2016). To exit the fetal liver, pMacs/monocytes must pass through diaphragms in the fenestrae of liver sinusoidal endothelium (Rantakari et al., 2016). Once through the diaphragms, they travel through the vasculature to all embryonic tissues (except the brain, which is now isolated by the blood-brain barrier). These fetal liver-EMPs represent the second wave of macrophages during development.

Adding to the ontogenetic diversity, hematopoietic stem cells (HSCs) are generated by hemogenic endothelium, primarily in the dorsal aorta in the aorta-gonad-mesonephros region (Medvinsky and Dzierzak, 1996; Yokomizo and Dzierzak, 2010). Fetal HSCs represent a third developmental wave of macrophages contributing to tissue-resident macrophage pools (Sheng et al., 2015). They enter the fetal liver from E10.5, expand and differentiate into monocytic intermediates, and then colonize tissues and mature into tissue-resident macrophages (Kumaravelu et al., 2002; Kieusseian et al., 2012; Sheng et al., 2015). During embryogenesis, HSCs also populate the bone marrow and spleen, where they are maintained postnatally as progenitors that can generate a constant supply of monocytes that can be released into the circulation. These HSC-derived adult circulating monocytes contribute to tissue-resident macrophage pools during tissue homeostasis in specific organs, such as the heart and intestine (Bain et al., 2014; Epelman et al., 2014a). In cases of injury and/or inflammation, an organ will recruit additional circulating monocytes that may worsen or limit the damage inflicted on the tissue (Tsou et al., 2007; Li et al., 2008; Shi and Pamer, 2011; Seok et al., 2013).

Recently, another bone marrow-derived source of adult macrophage progenitors has been discovered (Audzevich et al., 2017). These cells are bi-phenotypic early pro-B cells that express both myeloid and lymphoid markers. Like HSC-derived monocytes, early pro-B cells exit the bone marrow, travel through the circulation, and contribute to certain tissue-resident macrophage populations (such as in the peritoneum, pleural cavity, and intestine) during tissue homeostasis and inflammation (Audzevich et al., 2017).

**Figure 1:**
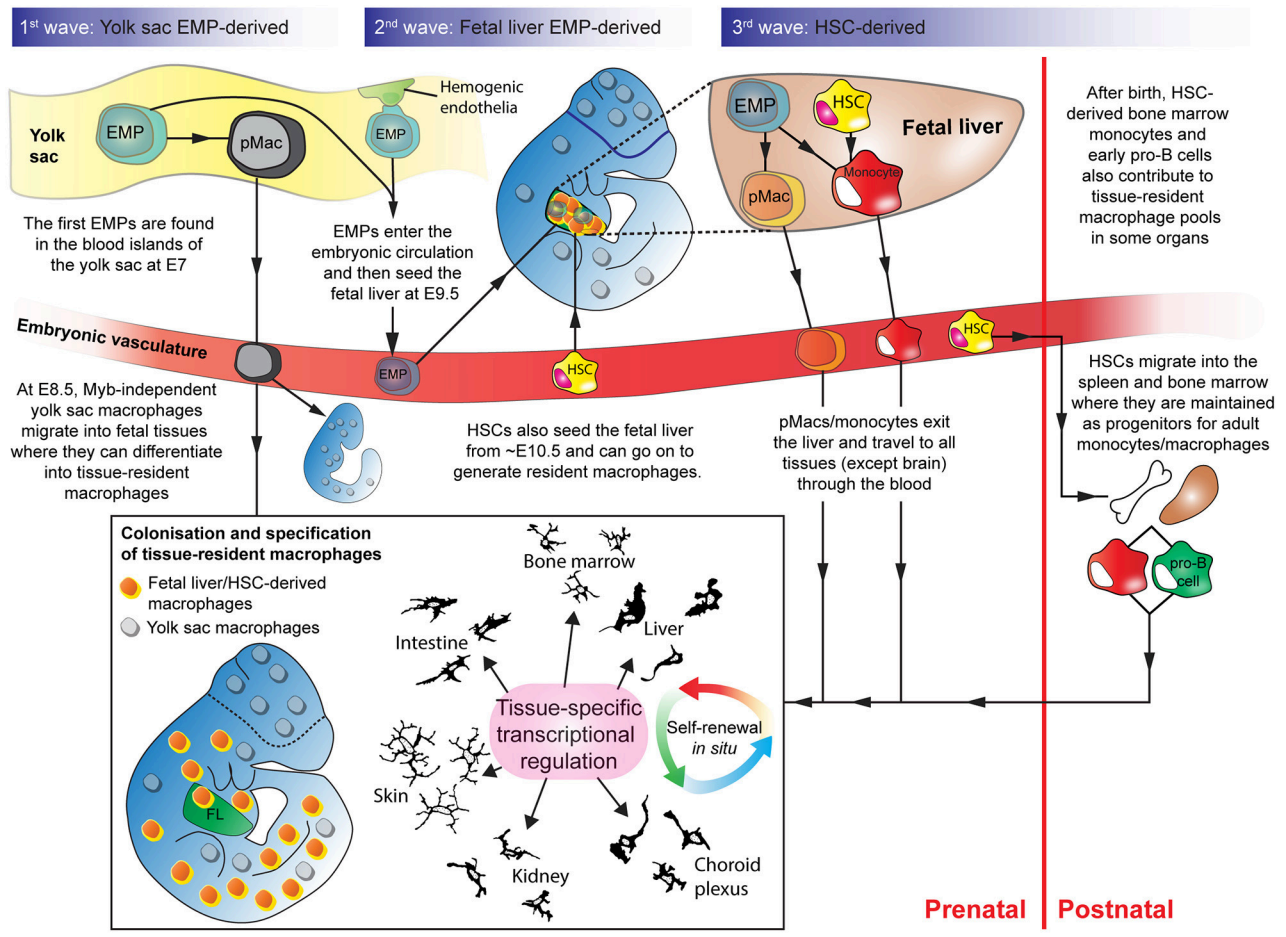
Overlapping waves of macrophage progenitor cells colonize the developing embryo. Wave 1: Yolk sac-derived erythro-myeloid progenitors (EMPs) differentiate into pMacs and migrate through the early embryo. Wave 2: EMPs, derived from hemogenic endothelium in the yolk sac, enter the fetal liver. In the fetal liver, EMPs expand and differentiate into pMacs and/or monocytes. pMacs/monocytes then egress from the fetal liver and travel through the vasculature into developing organs. Wave 3: Haematopoietic stem cells (HSCs) from the aorta-gonad-mesonephros region also enter the fetal liver and contribute to resident macrophage populations. Some HSCs migrate into the bone marrow and the spleen where they are maintained before being released into the bloodstream postnatally as circulating monocytes that can contribute to tissue-resident macrophage populations. Early pro-B cells are also released from the bone marrow in adulthood and can contribute to certain tissue-resident macrophage populations. Depending on where they engraft, macrophage progenitors start to express transcriptional regulators that define their genetic programme in a tissue-specific manner. EMP, erythro-myeloid progenitor; HSC, haematopoietic stem cell; pMac, pre-macrophage; Camera lucida drawings adapted, with permission, from illustrations by Perry (Gordon et al., 1988).

Thus, in most adult organs, tissue-resident macrophages derive from (1) fetal-generated macrophages that self-renew *in situ* (descending from the waves of progenitors) and (2) the engraftment of adult circulating macrophage progenitors (Figure 1).

## Origins of kidney macrophages

From the start of kidney development to the end of life, kidney-resident macrophages derive from all the progenitor waves described above: early yolk sac EMP-derived macrophages (Schulz et al., 2012; Hoeffel et al., 2015), fetal liver EMP-derived macrophages (Epelman et al., 2014a; Hoeffel et al., 2015), HSC-derived macrophages (Epelman et al., 2014a; Sheng et al., 2015), and adult bone marrow-derived circulating monocytes (Jang et al., 2013; de Cortie et al., 2014; Hoeffel et al., 2015; Sheng et al., 2015). The relative proportion of progenitors from each wave, however, changes dramatically throughout kidney development, adulthood, and in periods of disease.

The mouse metanephric (permanent) kidney begins to develop at ~E10.5, when the ureteric bud (the precursor of the collecting duct and ureter) emerges from the caudal end of the Wolffian/nephric duct in response to glial cell-line-derived neurotrophic factor (GDNF; Sainio et al., 1997). The ureteric bud invades the metanephric mesenchyme, a cell population comprised of nephron and stromal progenitors, and begins to branch. Throughout kidney development, signals from the metanephric mesenchyme induce further branching of the ureteric bud (Sainio et al., 1997; Majumdar et al., 2003; Costantini and Shakya, 2006). Simultaneously, ureteric bud branching induces nephron formation (Carroll et al., 2005) and guides vascular patterning (Munro et al., 2017).

**Figure 2:**
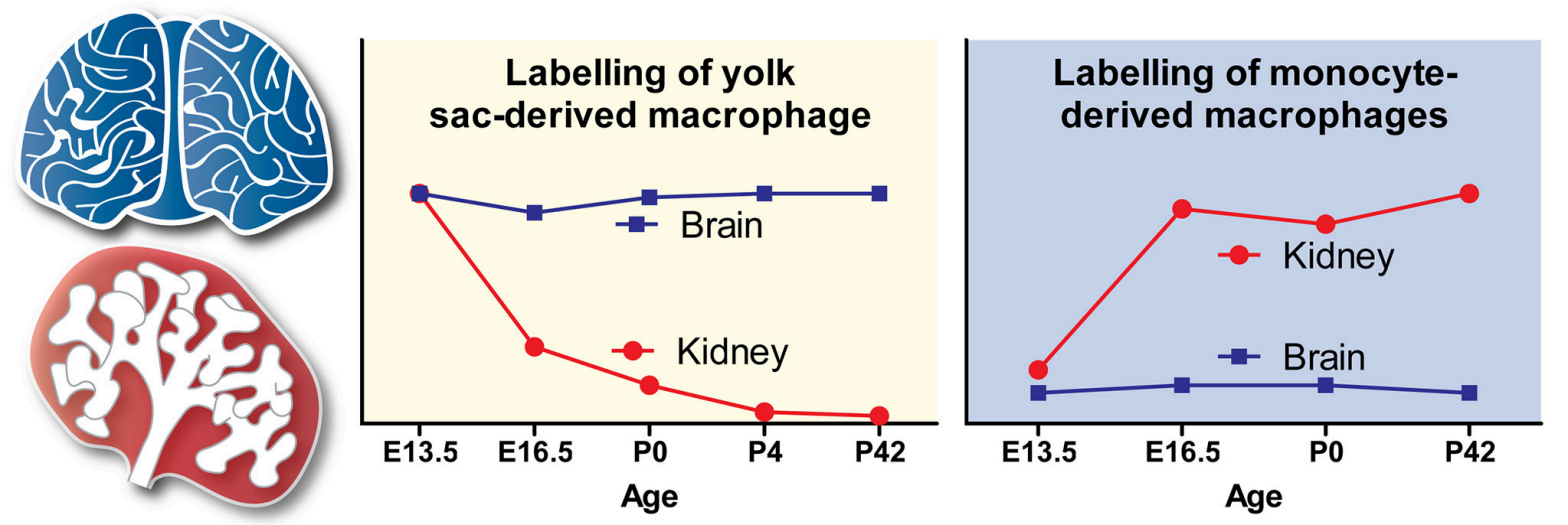
Contribution of yolk sac-derived and monocyte-derived macrophages to the embryonic and postnatal kidney. The trends shown in the yolk sac-derived and monocyte-derived macrophage graphs are based on fate-mapping experiments by Hoeffel et al. (2015). Trends for the origins of brain macrophages (microglia), which are yolk sac-derived, are shown as a comparison. Adapted with permission from Hoeffel et al. (2015).

There are no data regarding macrophage origins in the E10.5-E12 mouse kidney, possibly because so few macrophages are present in the kidney at this early developmental stage (Rae et al., 2007). At E12.5, however, flow cytometry analyses have revealed that kidney macrophages are yolk-sac EMP-derived (CD45^+^CD11b^lo^F4/80^hi^Ly6C^−^ cells). At this stage, no monocytes (CD45^+^CD11b^hi^F4/80^lo^Ly6C^+^ cells) are present within the kidney (Hoeffel et al., 2015). In fate-mapping studies with timed injections of hydroxytamoxifen (4′OHT) into tamoxifen-inducible *Runx1*^Cre/EYFP^ and *Csf1r*^Cre/EYFP^ mice (at E7.5 and E8.5, respectively; to label yolk sac macrophages and their progeny), the percentage of yolk sac-derived macrophages in the kidney was found to decrease exponentially from E13.5 to postnatal week 6 (Hoeffel et al., 2015). Runt-related transcription factor 1 (Runx1) is expressed by yolk sac-derived macrophage progenitors as they bud from hemogenic endothelium, and induction of labeling at E7.5 in *Runx1*^Cre/EYFP^ mice leads to specific EYFP-labeling of these cells (for details, see Hoeffel and Ginhoux, 2015). Colony stimulating factor 1 receptor (Csf1r) is expressed by all yolk sac-derived macrophage progenitors, and induction of labeling at E8.5 in *Csf1r*^Cre/EYFP^ mice leads to exclusive EYFP-labeling of these cells (for details, see Epelman et al., 2014b; Hoeffel et al., 2015). These data agree with the findings of Epelman et al. (2014a), who used similar techniques (i.e., timed injections of 4′OHT into tamoxifen-inducible *Csf1r*^Cre/GFP^ mice at E8.5) to show that yolk sac-derived macrophages make a minimal contribution to the pool of kidney macrophages by postnatal week 10. As yolk sac-derived macrophages are highly proliferative (based on flow cytometry analysis of Fucci-reporter embryos), and do not exhibit high rates of apoptosis (based on Annexin-V labeling followed by flow cytometry analysis), it has been speculated that this rapid decrease in the proportion of yolk sac-derived macrophages must be explained by their dilution by the marked later arrival of fetal monocytes (Hoeffel et al., 2015; Figure 2).

Fetal monocytes begin to populate the mouse kidney by E13.5 (Epelman et al., 2014a; Hoeffel et al., 2015). Between E13.5 and E16.5, the proportion of monocyte-derived macrophages within the kidney progressively increases, and rapidly exceeds that of yolk sac-derived macrophages (Epelman et al., 2014a; Hoeffel et al., 2015). Indeed, multiple studies have shown that adult kidney macrophages are almost exclusively fetal monocyte-derived (Epelman et al., 2014a; Hoeffel et al., 2015; Sheng et al., 2015); however, it is not clear whether these monocytes are generated from EMPs, HSCs, or a combination of both sources.

The precise origins of monocyte-derived kidney macrophages have been explored using various fate-mapping mouse models. In support of a HSC-derived source of adult kidney macrophages, Sheng et al. (2015) generated a tamoxifen-inducible c-Kit^Cre/EYFP^ mouse strain to fate-map the progeny of HSCs (all HSCs express c-Kit and induction of labeling leads to specific EYFP-labeling of these cells; for details, see Sheng et al., 2015); by labeling cells at several time-points they concluded that adult kidney macrophages derive from the HSC-precursor wave, rather than from EMPs. However, as c-Kit is expressed by EMPs as well as HSCs (Gomez Perdiguero et al., 2015), it is unclear whether this mouse model can reliably distinguish HSC-progenitors from EMP-progenitors (Hoeffel and Ginhoux, 2015). In another study by Epelman et al. (2014a), fate-mapping experiments were performed using Flt3^Cre/GFP^ mice. Flt3 is expressed transiently by all HSCs (Boyer et al., 2011), so an assumption of this model is that any monocyte/macrophage that goes through a Flt3^+^ stage (Flt3^Cre/GFP+^) can be classed as HSC-derived, whereas those that do not go through a Flt3^+^ stage (Flt3^Cre/GFP−^) can be said to be HSC-independent. Epelman et al. (2014a) demonstrated that recombination rates driven by Flt3 in adult kidney macrophages were ~50%, suggesting that there is a large contribution from both HSC-derived monocytes and HSC-independent, EMP-derived, monocytes to the pool of kidney-resident macrophages (Epelman et al., 2014a). Lastly, Hoeffel et al. (2015) determined that adult kidney macrophages predominantly arise from late c-Myb^+^ EMP-derived fetal monocytes. After injecting 4′OHT at E8.5 in the tamoxifen-inducible *Runx1*^Cre/EYFP^ fate-mapping mouse model, EMPs and their progeny were efficiently labeled, while there was only minor labeling of HSCs. Many of the labeled EMPs reached the fetal liver, and a significant proportion of these cells converted into the monocytes and macrophages that ultimately colonize various developing organs such as the kidney (Hoeffel et al., 2015). Together, these studies suggest that both EMP- and HSC-derived monocytes enter the kidney to generate mature kidney macrophages. However, the relative contribution of each progenitor type remains unclear. To explain the incongruent conclusions presented by these studies, further analyses of the specific drawbacks and limitations of the various fate-mapping models are necessary (as reviewed by Hoeffel and Ginhoux, 2015).

In adulthood, bone marrow-derived circulating monocytes colonize the healthy kidney at low levels (Sheng et al., 2015). When the kidney becomes diseased/inflamed/injured, monocyte infiltration dramatically increases, as has been shown in multiple experiments where the engraftment of bone-marrow derived cells was assessed in irradiated mice (Jang et al., 2013; de Cortie et al., 2014; Hoeffel et al., 2015).

In summary, kidney macrophage origins are diverse: the early kidney is colonized by yolk sac-derived macrophages, but the resident macrophages in the early postnatal kidney are predominantly derived from EMP- and HSC-derived monocytic precursors (Figure 2). These fetal-generated macrophages self-maintain throughout adulthood and are only partially replaced by bone marrow-derived circulating monocytes.

## What dictates kidney macrophage origins?

Although macrophage origins clearly differ between organs (Schulz et al., 2012; Hashimoto et al., 2013; Epelman et al., 2014a; Hoeffel et al., 2015), the origin of a tissue-resident macrophage does not seem to play a large role in determining its lifespan or functions (van de Laar et al., 2016; Guilliams and Scott, 2017). This begs the question, “why do macrophage origins differ?”

A recent hypothesis proposed to explain the differences in macrophage origins between tissues is the niche competition model, as proposed by Guilliams and Scott (2017). In this model, macrophage precursors populate a niche depending on its *accessibility*, its *availability*, and macrophage *precursor plasticity*. As macrophage precursors are extremely plastic (van de Laar et al., 2016; Guilliams and Scott, 2017), the question about macrophage ontogeny comes down to niche *accessibility* (e.g., can macrophage precursors access the organ via the circulation?) and *availability* (e.g., are there unoccupied niches in this organ for macrophage progenitors to exploit?).

Because the kidney is *accessible* to macrophage precursors via the systemic circulation throughout most of embryonic development (from ~E11.5; Munro et al., 2017) and adult life, it is unsurprising that kidney-resident macrophages derive from multiple waves of progenitors (Schulz et al., 2012; Ginhoux et al., 2016). In contrast, other organs, most notably the brain, become inaccessible to blood-borne cells during development: macrophage entry into the brain becomes restricted due to the establishment of the blood-brain-barrier and, consequently, brain macrophages (microglia) derive solely from the early yolk sac macrophages (Alliot et al., 1999; Hoeffel et al., 2015; Figure 2).

**Figure 3:**
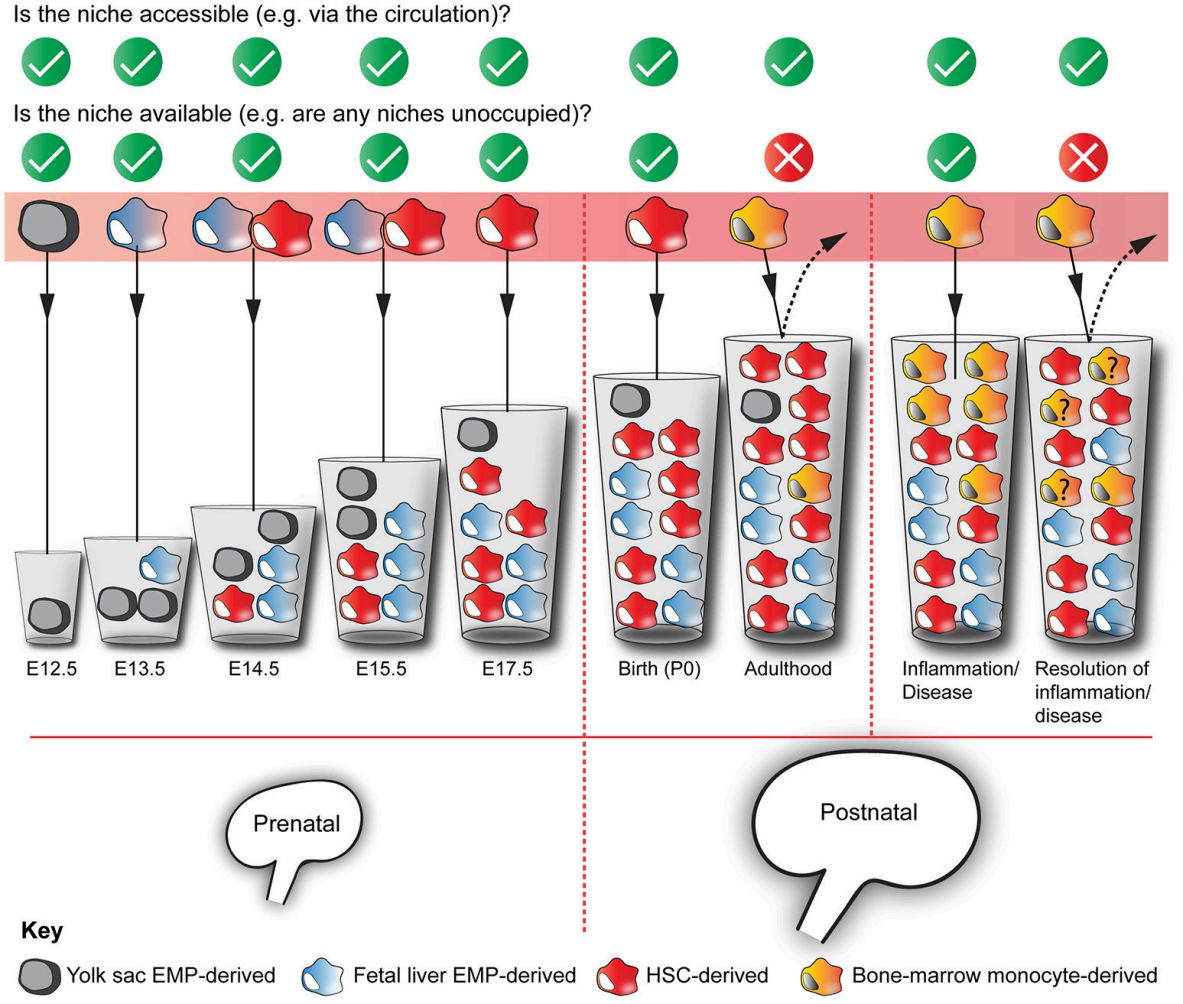
Multiple origins of kidney macrophages. Murine kidneys contain macrophages that are derived from multiple sources, with their relative proportions fluctuating throughout development and adulthood. Based on the niche competition hypothesis of macrophage origins (Guilliams and Scott, 2017), we argue that the mixed ontogeny of kidney macrophages is the result of kidney niches being both *accessible* and *available* to macrophage precursors throughout kidney development and phases of adulthood. The “**?**” in some of the yellow bone marrow-derived monocytes denotes that we do not know whether these cells are maintained in the kidney after the resolution of inflammation/disease. The increasing volumes of the cups represent the increasing capacity of the kidney to house macrophages.

As the kidney enlarges developmentally, its capacity for macrophage niches, and therefore its *availability* to house macrophages, will increase (Figure 3). Consequently, macrophages will colonize and fill these “developmental macrophage niches” as the kidney matures (from E10.5-P4; Short and Smyth, 2016). As kidney development ends, macrophage niches become fully occupied and, as a result, the macrophages that become resident within the kidney are predominantly embryonically/neonatally-derived (Epelman et al., 2014a; Hoeffel et al., 2015; Sheng et al., 2015). As these macrophages are long-lived and self-renew *in situ*, few circulating monocytes are provided with the opportunity to engraft within the kidney in adulthood (Figure 3). An exception to this might be during renal disease/inflammation/injury when niches become temporarily available due to the drainage of macrophages from the kidney via the lymphatics (Lan et al., 1993) and surges of macrophage-recruiting chemokines (Petrovic-Djergovic et al., 2015; Figure 3).

As well as being *accessible* and *available* to macrophages, an organ must produce signals to recruit macrophages. Signaling through Cx3c chemokine receptor 1 (Cx3cr1), the receptor for chemokine Cx3c motif ligand 1 (Cx3cl1), is important for macrophage migration and colonization of the early embryo (Imai et al., 1997; Mass et al., 2016). Caudal tissues, limbs, and the head of *Cx3cr1*-deficient embryos have decreased numbers of macrophages at E9.5 and E10.5 (Mass et al., 2016). However, most organs (lung, liver, and brain) in *Cx3cr1*^−/−^ embryos have normal macrophage numbers by E14.5 and in adulthood (relative to *Cx3cr1*^+/−^ littermates). Conversely, kidneys of *Cx3cr1*^−/−^ embryos have greatly diminished macrophage numbers at E14.5 and in adulthood (relative to *Cx3cr1*^+/−^ littermates; Mass et al., 2016). In agreement, Lionakis et al. (2013) also found that adult kidneys of *Cx3cr1*^−/−^ mice have reduced macrophage numbers. Moreover, kidneys of *Cx3cr1*^−/−^ mice recruited fewer macrophages in response to systemic infection with the fungus *Candida albicans* (relative to *Cx3cr1*^+/+^ mice). In *Cx3cr1*-deficient mice, this fungus was cleared from all organs tested apart from the kidney. Consequently, *Cx3cr1*^−/−^ mice uniformly succumbed to infection due to uncontrolled fungal proliferation in the kidney and renal failure (Lionakis et al., 2013). These data indicate that the Cx3cl1/Cx3cr1 signaling pathway is uniquely important for the recruitment of macrophages to the kidney during development, at steady state, and in response to infection.

## Specification of kidney macrophages

The expression of transcriptional regulators in a tissue-resident macrophage is regulated in a tissue-specific manner (Lavin et al., 2014; Amit et al., 2016; Mass et al., 2016). This regulation results in macrophages being specialized according to the needs of their specific organ of residence. Bulk RNA-seq data have identified candidate transcriptional regulators that might act to control kidney macrophage programming and differentiation (Mass et al., 2016).

In comparison to those in other developing organs (i.e., brain, liver, skin, and lung), kidney macrophages exhibit increased expression of the transcriptional regulators aryl hydrocarbon receptor (Ahr), nuclear factor of activated T cells 1 and 2 (Nfatc1 and Nfatc2), and interferon regulatory factor 9 (Irf9) (Mass et al., 2016). Ahr influences macrophage activation and production of nitric oxide and arginine (Climaco-Arvizu et al., 2016). Irf9 also influences macrophage activation (Ganta et al., 2017). Nfatc1 and Nfatc2 are involved in macrophage cytokine expression and the inflammatory response (Minematsu et al., 2011; Elloumi et al., 2012). However, although more highly expressed in kidney-resident than other resident macrophages, the effects of these transcriptional regulators on kidney macrophage specification and function have not been investigated.

**Figure 4:**
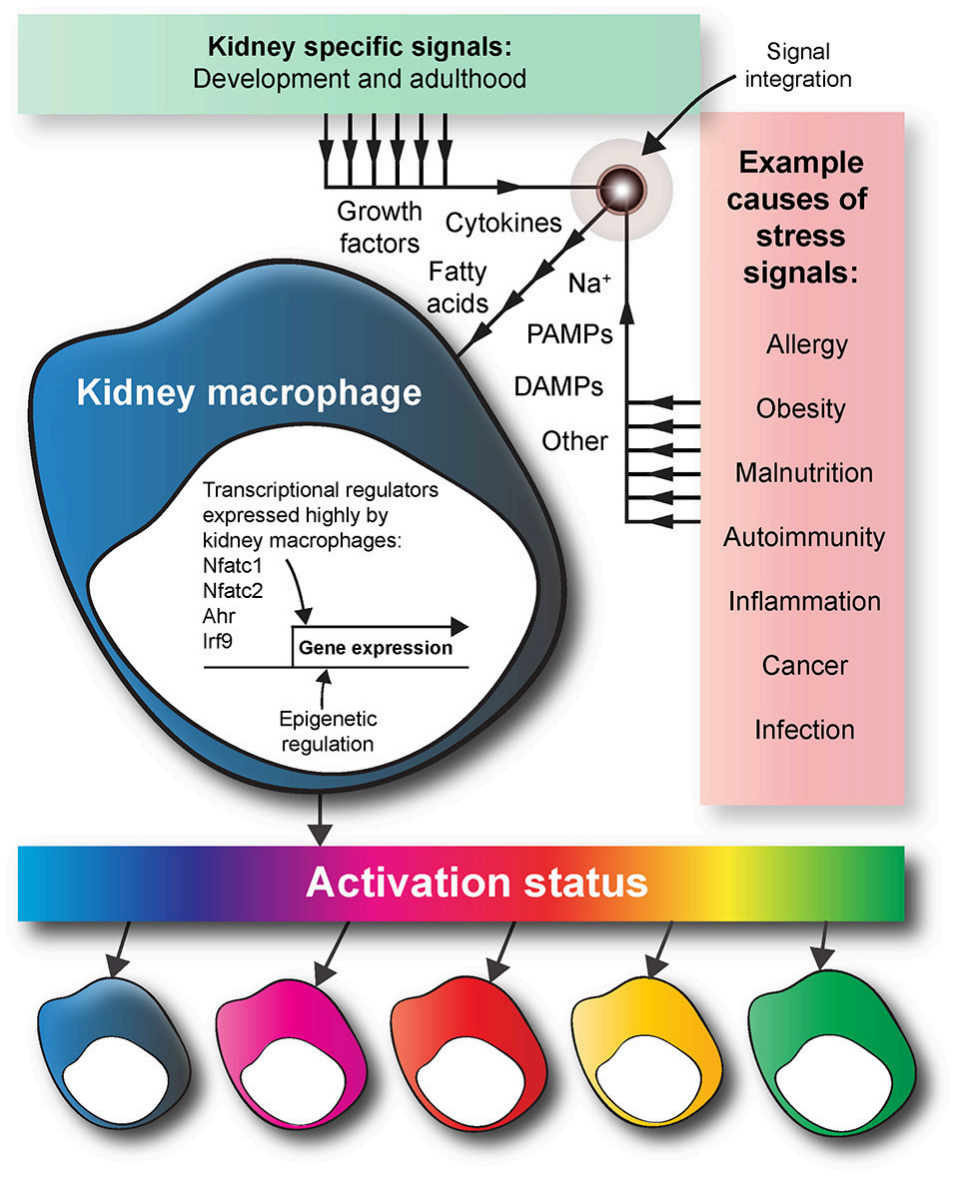
Kidney macrophage specification. During development, the tissue-specific signals that kidney macrophages are exposed to provoke the expression of a unique array of transcriptional regulators. Compared to other tissue-resident macrophages, kidney macrophages have increased expression of transcriptional regulators such as Nfatc1, Nfatc2, Ahr, and Irf9. In adulthood, macrophages are exposed to various exogenous stress signals because of factors such as disease, diet, and infection. Based on the multidimensional model of macrophage activation, a macrophage is specified by the integrated effects of the endogenous and exogenous signals within its micro-anatomical site. The macrophage colors are used to show the heterogeneity of macrophage activation status in response to endogenous and exogenous signals in the kidney. DAMPS, damage-associated molecular pattern molecules; PAMPS, pathogen-associated molecular pattern molecules.

A complication regarding the study of macrophage specification is the heterogeneity of macrophages, even within a single organ. In the adult kidney, there are at least five types of tissue-resident macrophages (Kawakami et al., 2013). Based on the multidimensional model of macrophage activation, macrophage specification depends on the integration of the environmental signals to which it is exposed, rather than being based on macrophage ontogeny (Ginhoux et al., 2016). The chemokine environment within the kidney will not be spatially uniform, and the micro-anatomical site of a macrophage will dictate its specification. For example, a macrophage is not exposed to identical signals in the renal cortex and the renal medulla (Berry et al., 2017). Furthermore, exogenous stress signals, such as small immune complexes (Stamatiades et al., 2016) that are carried into the kidney via the blood will also control macrophage specification in an environment-specific manner (Figure 4). Future studies should investigate how the integration of endogenous and exogenous signals dictates the phenotypes and functions of kidney macrophages in development and adulthood.

## Functions of macrophages in kidney development

While numerous reviews have detailed the functions of kidney macrophages in the healthy and diseased adult kidney (Rogers et al., 2014; Cao et al., 2015; Guiteras et al., 2016), there are few descriptions of the functions of kidney macrophages in renal development. In other developing organs, macrophages are critical in processes such as branching morphogenesis, angiogenesis, and the clearance of dead cells (Gouon-Evans et al., 2000; Kawane et al., 2003; Pollard, 2009; DeFalco et al., 2014; Sathi et al., 2017). It is now becoming clear that these processes are also facilitated by kidney macrophages during renal organogenesis.

## Cell death and clearance

An appropriate balance between cell death, survival, and proliferation is crucial for organ growth and remodeling during development (Penaloza et al., 2006). Cell death in the developing kidney peaks at E14.5 (0.25% of cells are apoptotic) and then drops considerably after birth (by postnatal day 14, only 0.06% of cells are apoptotic; Foley and Bard, 2002). Apoptosis during kidney development may facilitate the reciprocal signaling between the ureteric bud and nephron progenitor cells (Coles et al., 1993; Foley and Bard, 2002; Stewart and Bouchard, 2011). Inhibition of apoptosis in kidney explants, via blockade of caspase −3 and −9, reduces branching morphogenesis and nephrogenesis (Araki et al., 1999, 2003). The cellular debris released from these apoptotic cells is cleared via a process termed efferocytosis (Latin for “to take to the grave” or “to bury”).

During renal development, efferocytosis is carried out by kidney macrophages (Camp and Martin, 1996; Erdösová et al., 2002). “Find me” chemokine signals and “eat me” cell surface signals that are expressed by dying cells attract macrophages to phagocytose them (Truman et al., 2008; Nagata et al., 2010), resulting in the degradation of their cellular components (A-Gonzalez and Hidalgo, 2014). Where efferocytosis is defective, the innate immune system becomes activated, which leads to inflammation (Green et al., 2016). This can have negative consequences on a developing organ; indeed, in the developing thymus, it impairs thymic organogenesis, reducing thymocyte number, and thymus size (Kawane et al., 2003). However, the consequences of impaired efferocytosis in the developing kidney have not been investigated.

## Ureteric bud branching morphogenesis

Macrophages contribute to branching morphogenesis in the developing lung, mammary gland, and submandibular gland (Gouon-Evans et al., 2000; Pollard, 2009; Jones et al., 2013; Sathi et al., 2017). Evidence suggests that kidney macrophages similarly contribute to ureteric bud branching in the kidney (Rae et al., 2007; Muthukrishnan et al., 2017).

The addition of colony stimulating factor-1 (Csf-1) accelerates growth and ureteric bud branching in cultured kidney explants (Rae et al., 2007). Binding of the Csf-1 ligand to its membrane receptor, Csf1r, results in the activation of the Csf1r pathway and the stimulation of macrophage proliferation, survival, and differentiation (Dai et al., 2002; Mouchemore and Pixley, 2012). Macrophage numbers were increased in the Csf-1 treated kidney explants and their expression profile was consistent with them being alternatively activated, pro-proliferative M2 macrophages (Rae et al., 2007). Although the M1/M2 model of macrophage activation is imperfect (Murray et al., 2014), an M2-like activation status is associated with trophic/tissue remodeling macrophages (Mantovani et al., 2004; Marchetti et al., 2011). However, Rae et al. (2007) did not establish whether the trophic effects on kidney growth and ureteric bud branching were the result of the increased macrophage numbers, the alternative activation status of the macrophages, the exogenous addition of Csf-1 *per se*, or a combination of these possibilities. In support of the proposed trophic role of Csf-1 on kidney development, *in vivo* treatment of neonatal mice with Csf-1 resulted in the development of larger, heavier kidneys (Alikhan et al., 2011); as with the kidney explants, this growth was associated with increased numbers of tissue macrophages. Correspondingly, *Csf1r*-null mice have lighter kidneys compared to wild type mice (Alikhan et al., 2011) and male mice injected with an anti-Csf1r blocking antibody 3× weekly for 6 weeks have decreased kidney:body weight ratios (Sauter et al., 2014).

Kidney macrophages may also directly stimulate ureteric bud branching during development. Populations of nephron progenitor cells that cap ureteric bud tips (Reinhoff, 1922) secrete glial cell line-derived neurotrophic factor (Gdnf) to promote branching morphogenesis via activation of Ret receptor tyrosine kinase (Ret) on the membrane of ureteric bud epithelia (Schuchardt et al., 1994; Sainio et al., 1997). Following experimental ablation of nephron progenitor cells, macrophages compensate for their loss by localizing around ureteric bud tips and secreting Gdnf to maintain ureteric bud branching (Muthukrishnan et al., 2017). Whether macrophages play a similar role to directly stimulate ureteric bud branching during normal kidney development is currently unknown.

## Nephron formation

In the developing kidney, many macrophages are found near renal tubules (Rae et al., 2007). A direct role of macrophages in facilitating nephron formation has not been described during normal renal organogenesis, but, as with ureteric bud branching, Csf-1 treated kidney explants developed greater numbers of nephrons (Rae et al., 2007). Furthermore, macrophages are recruited to the nephrogenic zone (the site of nephron formation) when nephron progenitor cells are experimentally ablated where they stimulate nephron progenitor proliferation (Muthukrishnan et al., 2017). However, the relevance of this experimental model to normal kidney development is unknown.

As nephron formation is unique to the kidneys (pronephros, mesonephros, and metanephros), it is difficult to relate macrophage functions in other developing mammalian organs with the creation of new nephrons. Here, some invertebrate species, such as the fruit fly (*Drosophila melanogaster*), are providing insights. In *D. melanogaster*, the Malpighian tubules perform the function of the kidney and hemocytes are analogous to macrophages. Hemocytes deposit type IV collagen around the developing renal tubules, which sensitizes tubular cells to the BMP ligand, Decapentaplegic (Dpp; Bunt et al., 2010). Without hemocytes, or collagen IV, failure of BMP signaling leads to the misrouting of the anterior Malpighian tubules (Bunt et al., 2010). Whether this process is conserved in the mammalian kidney has not been investigated, but the nephron tubular basement membrane is rich in collagen IV (Abrahamson and Leardkamolkarn, 1991) and both human and murine macrophages can facilitate the deposition of almost every type of collagen (Vaage and Lindblad, 1990; Schnoor et al., 2008).

## Blood and lymphatic vessel development

Macrophages facilitate the vascularization of many developing organs (Fantin et al., 2010; Rymo et al., 2011; DeFalco et al., 2014). In the developing kidney, many macrophages localize around blood and lymphatic vessels (Rae et al., 2007; Lee et al., 2011), but their interactions have not been examined.

In angiogenesis, functional blood vessels form through a two-step process: endothelial tip cells first sprout from pre-existing vessels and then secondly, fuse with other blood vessels (anastomosis). Macrophages facilitate endothelial anastomosis in development (Fantin et al., 2010) and in response to vascular rupture (Liu et al., 2016). They do this by directly adhering to endothelia before generating mechanical traction forces to pull vessels together (Liu et al., 2016).

Moreover, macrophages are sensitive to low oxygen levels, and can promote angiogenesis in response to hypoxia (Cattin et al., 2015). When oxygen levels are low, signaling via the p110γ isoform of phosphoinositide 3-kinase (PI3K), which is predominantly expressed in macrophages, stabilizes hypoxia-inducible transcription factor 1/2-alpha (HIF1α and HIF2α; Joshi et al., 2014). This results in HIF1/2α translocation to the macrophage nucleus, where it binds to hypoxia-responsive elements to induce the expression of pro-angiogenic genes such as VEGF (Joshi et al., 2014). Macrophage-derived VEGF-A stimulates angiogenesis by promoting endothelial proliferation and migration to resolve hypoxia (Cattin et al., 2015). Hypoxia plays a role in developmental angiogenesis in the kidney (Abrahamson, 2009), and future studies should investigate whether kidney macrophages respond to hypoxia by guiding angiogenesis.

A subset of macrophages in the developing kidney express lymphatic vessel endothelial hyaluronan receptor 1 (Lyve-1; Lee et al., 2011). Lyve-1 is an endocytic receptor for hyaluronan that is also highly expressed by lymphatic endothelia. Lyve-1^+^ macrophages are reportedly pro-angiogenic and pro-lymphangiogenic (Cho et al., 2007; Harvey and Gordon, 2012). Lee et al. (2011) demonstrated that Lyve-1^+^ lymphatic endothelia are closely associated with Lyve-1^+^ macrophages in the developing kidney, and they speculated that the latter facilitate lymphatic development during renal organogenesis. This idea is supported by evidence that macrophages can express VEGF-C to stimulate lymphangiogenesis (Kerjaschki, 2005; Alishekevitz et al., 2016) and that macrophages can act as lymphatic endothelial progenitors that integrate into sprouting lymphatic vessels (Ran and Montgomery, 2012). Furthermore, a subtype of VEGF-C-expressing interstitial macrophages has previously been associated with lymphangiogenesis in human kidney transplants (Kerjaschki et al., 2004). Based on these data, kidney macrophages could play a role in stimulating lymphatic development in a VEGF-C dependent and/or independent manner.

**Figure 5:**
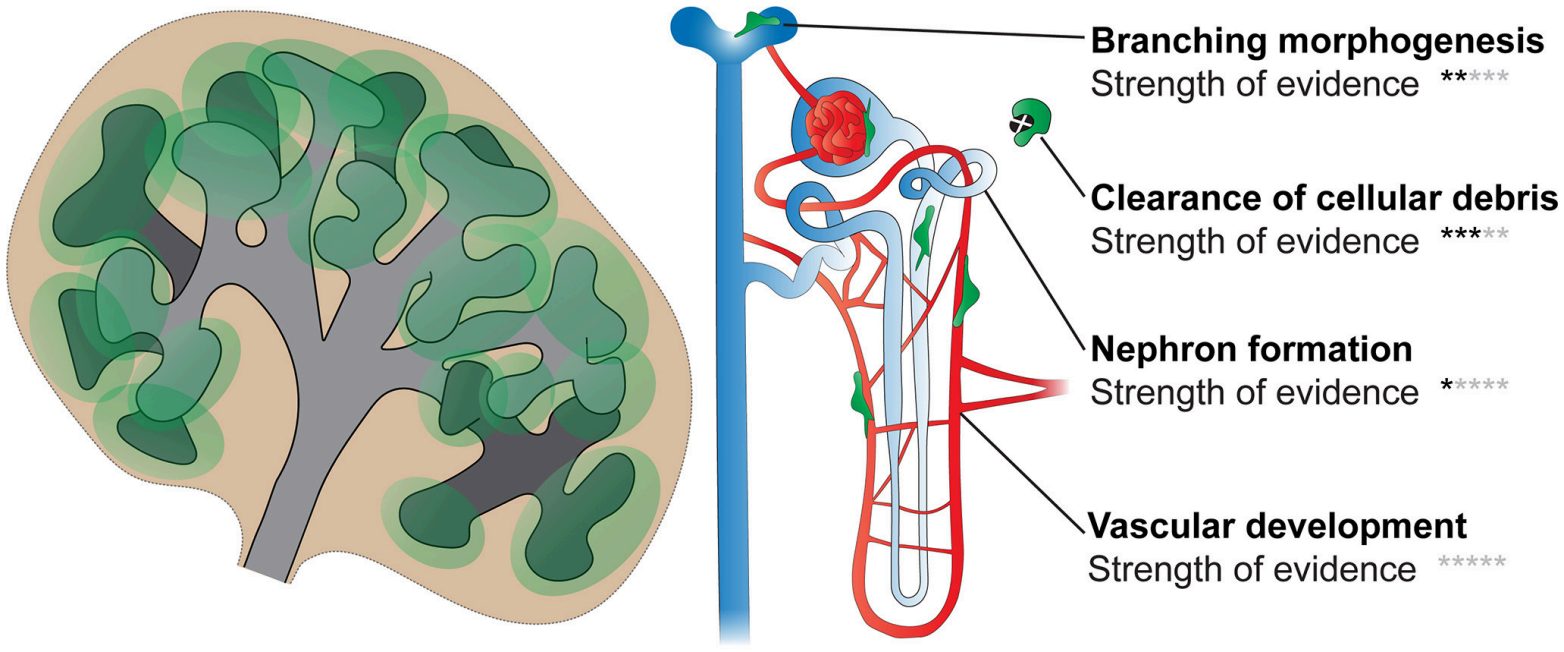
Functions of macrophages in kidney development. Processes including branching morphogenesis, cell proliferation/death (and clearance of cellular debris), nephron formation, and vascular development (blood and lymphatic) are important in renal development. Few studies have directly investigated the roles of kidney macrophages in these processes; however, based on available evidence, it is likely that macrophages will facilitate most, if not all, of these processes. Black stars indicate the strength of evidence that macrophages function in each process in kidney development (0 stars, no direct evidence; 5 stars, very strong evidence).

Although it seems likely that kidney macrophages will facilitate the processes described above, more studies are undoubtedly required before we fully appreciate the functional requirements of macrophages during renal development (Figure 5).

## Conclusions and future directions

In many organs, including the kidney, tissue-resident macrophages are predominantly derived from embryonic macrophages. In the kidney, these embryonic macrophages are mainly generated from fetal monocytes, which are specified and self-renew *in situ* throughout development and adulthood. Kidney macrophages are endowed with a unique genetic programme that allows them to promote normal renal organogenesis and to maintain the health and function of the adult organ. It will be extremely challenging to fully characterize the factors involved in kidney macrophage specification, and to understand how this dictates the functions of a given macrophage. Nevertheless, this is a challenge that should be faced, as it may expose new therapeutic opportunities to prevent and treat a range of developmental and pathological conditions.

To better understand kidney macrophage specification, future studies should utilize single-cell transcriptomic technologies to spatiotemporally classify the phenotypes of distinct macrophage populations in the developing kidney. By identifying clusters of macrophages with distinct gene expression patterns, and determining where they localize in the kidney, it may be possible to link environment-specific signals to the phenotypes and functions of a macrophage.

As macrophages can promote kidney growth, they could potentially be used therapeutically to assist renal development in babies at risk of preterm birth. Increasing macrophage recruitment to the developing kidney through treatment with chemokines such as Csf-1 and/or Cx3cr1 could conceivably promote kidney growth and nephron endowment. However, macrophages can also promote abnormalities in kidney development, such as cyst formation (Karihaloo et al., 2011), and their recruitment has previously been associated with disease progression in Wilms tumor (Liou et al., 2013); in these cases, the elimination of macrophages may be more therapeutic. Accordingly, macrophage-based therapies for developmental abnormalities will have to be context-dependent and will necessitate an in-depth understanding of kidney macrophage specification and function.

The inflammatory properties of kidney macrophages and recruited circulating monocytes are implicated in the initiation and progression of injury and scarring in the adult kidney as well as kidney regeneration and healing (reviewed in Rogers et al., 2014). Although beyond the scope of this review, it is of interest that the recruited monocyte-derived macrophages, but not kidney-resident macrophages, are pro-fibrotic in the obstructed kidney (Lin et al., 2009). In addition, commonalities exist between macrophages in the healing adult kidneys and the developing kidney (Alikhan et al., 2011). As well as the therapeutic potential of using macrophages to mitigate abnormal kidney development, studying the specification, and function of kidney macrophages in development may also provide new therapeutic/preventative options to minimize renal complications in adulthood.

## Author contributions

DM conceptualized the review, wrote the manuscript, and prepared the figures. JH reviewed and edited the manuscript.

### Conflict of interest statement

The authors declare that the research was conducted in the absence of any commercial or financial relationships that could be construed as a potential conflict of interest.

## References

[B1] Abrahamson D. R. (2009). Development of kidney glomerular endothelial cells and their role in basement membrane assembly. Organogenesis 5, 275–287. 10.4161/org.0.7577 19568349 PMC2659369

[B2] Abrahamson D. R. Leardkamolkarn V. (1991). Development of kidney tubular basement membranes. Kidney Int. 39, 382–393. 10.1038/ki.1991.50 2062031

[B3] Gonzalez N. Hidalgo A. (2014). Nuclear receptors and clearance of apoptotic cells: stimulating the macrophage's appetite. Front. Immunol. 5:211 10.3389/fimmu.2014.00211 24860573 PMC4026730

[B4] Ajami B. Bennett J. L. Krieger C. Tetzlaff W. Rossi F. M. (2007). Local self-renewal can sustain CNS microglia maintenance and function throughout adult life. Nat. Neurosci. 10, 1538–1543. 10.1038/nn2014 18026097

[B5] Alikhan M. A. Jones C. V. Williams T. M. Beckhouse A. G. Fletcher A. L. Kett M. M.. (2011). Colony-stimulating factor-1 promotes kidney growth and repair via alteration of macrophage responses. Am. J. Pathol. 179, 1243–1256. 10.1016/j.ajpath.2011.05.037 21762674 PMC3157188

[B6] Alishekevitz D. Gingis-Velitski S. Kaidar-Person O. Gutter-Kapon L. Scherer S. D. Raviv Z.. (2016). Macrophage-induced lymphangiogenesis and metastasis following paclitaxel chemotherapy is regulated by VEGFR3. Cell Rep. 17, 1344–1356. 10.1016/j.celrep.2016.09.083 27783948 PMC5098117

[B7] Alliot F. Godin I. Pessac B. (1999). Microglia derive from progenitors, originating from the yolk sac, and which proliferate in the brain. Brain Res. Dev. Brain Res. 117, 142–152. 10.1016/S0165-3806(99)00113-3 10567732

[B8] Amit I. Winter D. R. Jung S. (2016). The role of the local environment and epigenetics in shaping macrophage identity and their effect on tissue homeostasis. Nat. Immunol. 17, 18–25. 10.1038/ni.3325 26681458

[B9] Araki T. Hayashi M. Nakanishi K. Morishima N. Saruta T. (2003). Caspase-9 takes part in programmed cell death in developing mouse kidney. Nephron Exp. Nephrol. 93, e117–e124. 10.1159/000069552 12660414

[B10] Araki T. Saruta T. Okano H. Miura M. (1999). Caspase activity is required for nephrogenesis in the developing mouse metanephros. Exp. Cell Res. 248, 423–429. 10.1006/excr.1999.4424 10222134

[B11] Aschoff L. (1924). Das reticulo-endotheliale system. Ergeb. Inn. Med. Kinderheilkd. 26, 1–118. 10.1007/978-3-642-90639-8_1

[B12] Audzevich T. Bashford-Rogers R. Mabbott N. A. Frampton D. Freeman T. C. Potocnik A.. (2017). Pre/pro-B cells generate macrophage populations during homeostasis and inflammation. Proc. Natl. Acad. Sci. U.S.A. 114, E3954–E3963. 10.1073/pnas.1616417114 28461481 PMC5441795

[B13] Bain C. C. Bravo-Blas A. Scott C. L. Perdiguero E. G. Geissmann F. Henri S.. (2014). Constant replenishment from circulating monocytes maintains the macrophage pool in the intestine of adult mice. Nat. Immunol. 15, 929–937. 10.1038/ni.2967 25151491 PMC4169290

[B14] Berry M. R. Mathews R. J. Ferdinand J. R. Jing C. Loudon K. W. Wlodek E.. (2017). Renal sodium gradient orchestrates a dynamic antibacterial defense zone. Cell 170, 860–874. 10.1016/j.cell.2017.07.022 28803730

[B15] Boyer S. W. Schroeder A. V. Smith-Berdan S. Forsberg E. C. (2011). All hematopoietic cells develop from hematopoietic stem cells through Flk2/Flt3-positive progenitor cells. Cell Stem Cell 9, 64–73. 10.1016/j.stem.2011.04.021 21726834 PMC4103692

[B16] Bunt S. Hooley C. Hu N. Scahill C. Weavers H. Skaer H. (2010). Hemocyte-secreted type IV collagen enhances BMP signaling to guide renal tubule morphogenesis in *Drosophila*. Dev. Cell 19, 296–306. 10.1016/j.devcel.2010.07.019 20708591 PMC2941037

[B17] Camp V. Martin P. (1996). The role of macrophages in clearing programmed cell death in the developing kidney. Anat. Embryol. 194, 341–348. 10.1007/BF00198535 8896697

[B18] Cao Q. Harris D. C. Wang Y. (2015). Macrophages in kidney injury, inflammation, and fibrosis. Physiology 30, 183–194. 10.1152/physiol.00046.2014 25933819

[B19] Carroll T. J. Park J. S. Hayashi S. Majumdar A. McMahon A. P. (2005). Wnt9b plays a central role in the regulation of mesenchymal to epithelial transitions underlying organogenesis of the mammalian urogenital system. Dev. Cell 9, 283–292. 10.1016/j.devcel.2005.05.016 16054034

[B20] Cattin A. L. Burden J. J. Van Emmenis L. Mackenzie F. E. Hoving J. J. Garcia Calavia N.. (2015). Macrophage-induced blood vessels guide schwann cell-mediated regeneration of peripheral nerves. Cell 162, 1127–1139. 10.1016/j.cell.2015.07.021 26279190 PMC4553238

[B21] Cho C. H. Koh Y. J. Han J. Sung H. K. Jong Lee H. Morisada T.. (2007). Angiogenic role of LYVE-1-positive macrophages in adipose tissue. Circ. Res. 100, e47–e57. 10.1161/01.RES.0000259564.92792.93 17272806

[B22] Climaco-Arvizu S. Domínguez-Acosta O. Cabañas-Cortés M. A. Rodríguez-Sosa M. Gonzalez F. J. Vega L.. (2016). Aryl hydrocarbon receptor influences nitric oxide and arginine production and alters M1/M2 macrophage polarization. Life Sci. 155, 76–84. 10.1016/j.lfs.2016.05.001 27153778 PMC6300993

[B23] Cline M. J. Moore M. A. (1972). Embryonic origin of the mouse macrophage. Blood 39, 842–849. 5028525

[B24] Coles H. S. Burne J. F. Raff M. C. (1993). Large-scale normal cell death in the developing rat kidney and its reduction by epidermal growth factor. Development 118, 777–784. 8076517 10.1242/dev.118.3.777

[B25] Costantini F. Shakya R. (2006). GDNF/Ret signaling and the development of the kidney. Bioessays 28, 117–127. 10.1002/bies.20357 16435290

[B26] Dai X. M. Ryan G. R. Hapel A. J. Dominguez M. G. Russell R. G. Kapp S.. (2002). Targeted disruption of the mouse colony-stimulating factor 1 receptor gene results in osteopetrosis, mononuclear phagocyte deficiency, increased primitive progenitor cell frequencies, and reproductive defects. Blood 99, 111–120. 10.1182/blood.V99.1.111 11756160

[B27] de Cortie K. Russell N. S. Coppes R. P. Stewart F. A. Scharpfenecker M. (2014). Bone marrow-derived macrophages incorporate into the endothelium and influence vascular and renal function after irradiation. Int. J. Radiat. Biol. 90, 769–777. 10.3109/09553002.2014.920967 24797272

[B28] DeFalco T. Bhattacharya I. Williams A. V. Sams D. M. Capel B. (2014). Yolk-sac-derived macrophages regulate fetal testis vascularization and morphogenesis. Proc. Natl. Acad. Sci. U.S.A. 111, E2384–E2393. 10.1073/pnas.1400057111 24912173 PMC4060703

[B29] Elloumi H. Z. Maharshak N. Rao K. N. Kobayashi T. Ryu H. S. Mühlbauer M.. (2012). A cell permeable peptide inhibitor of NFAT inhibits macrophage cytokine expression and ameliorates experimental colitis. PLoS ONE 7:e34172. 10.1371/journal.pone.0034172 22479554 PMC3313977

[B30] Eom D. S. Parichy D. M. (2017). A macrophage relay for long-distance signaling during postembryonic tissue remodeling. Science 355, 1317–1320. 10.1126/science.aal2745 28209639 PMC5836293

[B31] Epelman S. Lavine K. J. Beaudin A. E. Sojka D. K. Carrero J. A. Calderon B.. (2014a). Embryonic and adult-derived resident cardiac macrophages are maintained through distinct mechanisms at steady state and during inflammation. Immunity 40, 91–104. 10.1016/j.immuni.2013.11.019 24439267 PMC3923301

[B32] Epelman S. Lavine K. J. Randolph G. J. (2014b). Origin and functions of tissue macrophages. Immunity 41, 21–35. 10.1016/j.immuni.2014.06.013 25035951 PMC4470379

[B33] Erdösová B. Hlávková L. Procházková J. Lichnovský V. (2002). Part of CD68+ macrophages in the clearence of apoptotic bodies in human metanephros. Biomed. Pap. Med. Fac. Univ. Palacky Olomouc Czech. Repub. 146, 41–45. 10.5507/bp.2002.008 12572894

[B34] Fantin A. Vieira J. M. Gestri G. Denti L. Schwarz Q. Prykhozhij S.. (2010). Tissue macrophages act as cellular chaperones for vascular anastomosis downstream of VEGF-mediated endothelial tip cell induction. Blood 116, 829–840. 10.1182/blood-2009-12-257832 20404134 PMC2938310

[B35] Foley J. G. Bard J. B. (2002). Apoptosis in the cortex of the developing mouse kidney. J. Anat. 201, 477–484. 10.1046/j.1469-7580.2002.00114.x 12489759 PMC1570997

[B36] Ganta V. C. Choi M. H. Kutateladze A. Fox T. E. Farber C. R. Annex B. H. (2017). A microRNA93-interferon regulatory factor-9-immunoresponsive gene-1-itaconic acid pathway modulates M2-like macrophage polarization to revascularize ischemic muscle. Circulation 135, 2403–2425. 10.1161/CIRCULATIONAHA.116.025490 28356443 PMC5503157

[B37] Ginhoux F. Schultze J. L. Murray P. J. Ochando J. Biswas S. K. (2016). New insights into the multidimensional concept of macrophage ontogeny, activation and function. Nat. Immunol. 17, 34–40. 10.1038/ni.3324 26681460

[B38] Godwin J. W. Pinto A. R. Rosenthal N. A. (2013). Macrophages are required for adult salamander limb regeneration. Proc. Natl. Acad. Sci. U.S.A. 110, 9415–9420. 10.1073/pnas.1300290110 23690624 PMC3677454

[B39] Gomez Perdiguero E. Geissmann F. (2013). Myb-independent macrophages: a family of cells that develops with their tissue of residence and is involved in its homeostasis. Cold Spring Harb. Symp. Quant. Biol. 78, 91–100. 10.1101/sqb.2013.78.020032 24122769

[B40] Gomez Perdiguero E. Klapproth K. Schulz C. Busch K. Azzoni E. Crozet L.. (2015). Tissue-resident macrophages originate from yolk-sac-derived erythro-myeloid progenitors. Nature 518, 547–551. 10.1038/nature13989 25470051 PMC5997177

[B41] Gordon S. (2008). Elie Metchnikoff: father of natural immunity. Eur. J. Immunol. 38, 3257–3264. 10.1002/eji.200838855 19039772

[B42] Gordon S. Perry V. H. Rabinowitz S. Chung L. P. Rosen H. (1988). Plasma membrane receptors of the mononuclear phagocyte system. J. Cell Sci. Suppl. 9, 1–26. 10.1242/jcs.1988.Supplement_9.1 3077135

[B43] Gouon-Evans V. Rothenberg M. E. Pollard J. W. (2000). Postnatal mammary gland development requires macrophages and eosinophils. Development 127, 2269–2282. 10804170 10.1242/dev.127.11.2269

[B44] Green D. R. Oguin T. H. Martinez J. (2016). The clearance of dying cells: table for two. Cell Death Differ. 23, 915–926. 10.1038/cdd.2015.172 26990661 PMC4987729

[B45] Guilliams M. Scott C. L. (2017). Does niche competition determine the origin of tissue-resident macrophages? Nat. Rev. Immunol. 17, 451–460. 10.1038/nri.2017.42 28461703

[B46] Guiteras R. Flaquer M. Cruzado J. M. (2016). Macrophage in chronic kidney disease. Clin. Kidney J. 9, 765–771. 10.1093/ckj/sfw096 27994852 PMC5162417

[B47] Harvey N. L. Gordon E. J. (2012). Deciphering the roles of macrophages in developmental and inflammation stimulated lymphangiogenesis. Vasc. Cell 4:15. 10.1186/2045-824X-4-15 22943568 PMC3444946

[B48] Hashimoto D. Chow A. Noizat C. Teo P. Beasley M. B. Leboeuf M.. (2013). Tissue-resident macrophages self-maintain locally throughout adult life with minimal contribution from circulating monocytes. Immunity 38, 792–804. 10.1016/j.immuni.2013.04.004 23601688 PMC3853406

[B49] Hoeffel G. Chen J. Lavin Y. Low D. Almeida F. F. See P.. (2015). C-Myb(+) erythro-myeloid progenitor-derived fetal monocytes give rise to adult tissue-resident macrophages. Immunity 42, 665–678. 10.1016/j.immuni.2015.03.011 25902481 PMC4545768

[B50] Hoeffel G. Ginhoux F. (2015). Ontogeny of tissue-resident macrophages. Front. Immunol. 6:486. 10.3389/fimmu.2015.00486 26441990 PMC4585135

[B51] Imai T. Hieshima K. Haskell C. Baba M. Nagira M. Nishimura M.. (1997). Identification and molecular characterization of fractalkine receptor CX3CR1, which mediates both leukocyte migration and adhesion. Cell 91, 521–530. 10.1016/S0092-8674(00)80438-9 9390561

[B52] Jang H. S. Kim J. I. Jung K. J. Kim J. Han K. H. Park K. M. (2013). Bone marrow-derived cells play a major role in kidney fibrosis via proliferation and differentiation in the infiltrated site. Biochim. Biophys. Acta 1832, 817–825. 10.1016/j.bbadis.2013.02.016 23466592

[B53] Jones C. V. Williams T. M. Walker K. A. Dickinson H. Sakkal S. Rumballe B. A.. (2013). M2 macrophage polarisation is associated with alveolar formation during postnatal lung development. Respir. Res. 14:41. 10.1186/1465-9921-14-41 23560845 PMC3626876

[B54] Joshi S. Singh A. R. Zulcic M. Durden D. L. (2014). A macrophage-dominant PI3K isoform controls hypoxia-induced HIF1α and HIF2α stability and tumor growth, angiogenesis, and metastasis. Mol. Cancer Res. 12, 1520–1531. 10.1158/1541-7786.MCR-13-0682 25103499

[B55] Karihaloo A. Koraishy F. Huen S. C. Lee Y. Merrick D. Caplan M. J.. (2011). Macrophages promote cyst growth in polycystic kidney disease. J. Am. Soc. Nephrol. 22, 1809–1814. 10.1681/ASN.2011010084 21921140 PMC3187181

[B56] Kasaai B. Caolo V. Peacock H. M. Lehoux S. Gomez-Perdiguero E. Luttun A.. (2017). Erythro-myeloid progenitors can differentiate from endothelial cells and modulate embryonic vascular remodeling. Sci. Rep. 7:43817. 10.1038/srep43817 28272478 PMC5341067

[B57] Kawakami T. Lichtnekert J. Thompson L. J. Karna P. Bouabe H. Hohl T. M.. (2013). Resident renal mononuclear phagocytes comprise five discrete populations with distinct phenotypes and functions. J. Immunol. 191, 3358–3372. 10.4049/jimmunol.1300342 23956422 PMC3808972

[B58] Kawane K. Fukuyama H. Yoshida H. Nagase H. Ohsawa Y. Uchiyama Y.. (2003). Impaired thymic development in mouse embryos deficient in apoptotic DNA degradation. Nat. Immunol. 4, 138–144. 10.1038/ni881 12524536

[B59] Kerjaschki D. (2005). The crucial role of macrophages in lymphangiogenesis. J. Clin. Invest. 115, 2316–2319. 10.1172/JCI26354 16138185 PMC1193892

[B60] Kerjaschki D. Regele H. M. Moosberger I. Nagy-Bojarski K. Watschinger B. Soleiman A.. (2004). Lymphatic neoangiogenesis in human kidney transplants is associated with immunologically active lymphocytic infiltrates. J. Am. Soc. Nephrol. 15, 603–612. 10.1097/01.ASN.0000113316.52371.2E 14978162

[B61] Kierdorf K. Erny D. Goldmann T. Sander V. Schulz C. Perdiguero E. G.. (2013). Microglia emerge from erythromyeloid precursors via Pu.1- and Irf8-dependent pathways. Nat. Neurosci. 16, 273–280. 10.1038/nn.3318 23334579

[B62] Kieusseian A. Brunet de la Grange P. Burlen-Defranoux O. Godin I. Cumano A. (2012). Immature hematopoietic stem cells undergo maturation in the fetal liver. Development 139, 3521–3530. 10.1242/dev.079210 22899849

[B63] Kumaravelu P. Hook L. Morrison A. M. Ure J. Zhao S. Zuyev S.. (2002). Quantitative developmental anatomy of definitive haematopoietic stem cells/long-term repopulating units (HSC/RUs): role of the aorta-gonad-mesonephros (AGM) region and the yolk sac in colonisation of the mouse embryonic liver. Development 129, 4891–4899. 12397098 10.1242/dev.129.21.4891

[B64] Lan H. Y. Nikolic-Paterson D. J. Atkins R. C. (1993). Trafficking of inflammatory macrophages from the kidney to draining lymph nodes during experimental glomerulonephritis. Clin. Exp. Immunol. 92, 336–341. 10.1111/j.1365-2249.1993.tb03401.x 8485918 PMC1554823

[B65] Lavin Y. Winter D. Blecher-Gonen R. David E. Keren-Shaul H. Merad M.. (2014). Tissue-resident macrophage enhancer landscapes are shaped by the local microenvironment. Cell 159, 1312–1326. 10.1016/j.cell.2014.11.018 25480296 PMC4437213

[B66] Lee H. W. Qin Y. X. Kim Y. M. Park E. Y. Hwang J. S. Huo G. H. (2011). Expression of lymphatic endothelium-specific hyaluronan receptor LYVE-1 in the developing mouse kidney. Cell Tissue Res. 343, 429–444. 10.1007/s00441-010-1098-x 21181199

[B67] Li J. J. Zhang Y. P. Yang P. Zeng H. S. Qian X. W. Zhang C. Y.. (2008). Increased peripheral circulating inflammatory cells and plasma inflammatory markers in patients with variant angina. Coron. Artery Dis. 19, 293–297. 10.1097/MCA.0b013e3282fd5c4e 18607165

[B68] Lin S. L. Castaño A. P. Nowlin B. T. Lupher M. L. Duffield J. S. (2009). Bone marrow Ly6Chigh monocytes are selectively recruited to injured kidney and differentiate into functionally distinct populations. J. Immunol. 183, 6733–6743. 10.4049/jimmunol.0901473 19864592

[B69] Lionakis M. S. Swamydas M. Fischer B. G. Plantinga T. S. Johnson M. D. Jaeger M.. (2013). CX3CR1-dependent renal macrophage survival promotes *Candida* control and host survival. J. Clin. Invest. 123, 5035–5051. 10.1172/JCI71307 24177428 PMC3859390

[B70] Liou P. Bader L. Wang A. Yamashiro D. Kandel J. J. (2013). Correlation of tumor-associated macrophages and clinicopathological factors in Wilms tumor. Vasc. Cell 5:5. 10.1186/2045-824X-5-5 23514200 PMC3610208

[B71] Liu C. Wu C. Yang Q. Gao J. Li L. Yang D.. (2016). Macrophages mediate the repair of brain vascular rupture through direct physical adhesion and mechanical traction. Immunity 44, 1162–1176. 10.1016/j.immuni.2016.03.008 27156384

[B72] Majumdar A. Vainio S. Kispert A. McMahon J. McMahon A. P. (2003). Wnt11 and Ret/Gdnf pathways cooperate in regulating ureteric branching during metanephric kidney development. Development 130, 3175–3185. 10.1242/dev.00520 12783789

[B73] Mantovani A. Sica A. Sozzani S. Allavena P. Vecchi A. Locati M.. (2004). The chemokine system in diverse forms of macrophage activation and polarization. Trends Immunol. 25, 677–686. 10.1016/j.it.2004.09.015 15530839

[B74] Marchetti V. Yanes O. Aguilar E. Wang M. Friedlander D. Moreno S.. (2011). Differential macrophage polarization promotes tissue remodeling and repair in a model of ischemic retinopathy. Sci. Rep. 1:76. 10.1038/srep00076 22355595 PMC3216563

[B75] Mass E. Ballesteros I. Farlik M. Halbritter F. Günther P. Crozet L.. (2016). Specification of tissue-resident macrophages during organogenesis. Science 353:6304. 10.1126/science.aaf4238 27492475 PMC5066309

[B76] McGrath K. E. Frame J. M. Fegan K. H. Bowen J. R. Conway S. J. Catherman S. C.. (2015). Distinct sources of hematopoietic progenitors emerge before HSCs and provide functional blood cells in the mammalian embryo. Cell Rep. 11, 1892–1904. 10.1016/j.celrep.2015.05.036 26095363 PMC4490098

[B77] McGrath K. E. Koniski A. D. Malik J. Palis J. (2003). Circulation is established in a stepwise pattern in the mammalian embryo. Blood 101, 1669–1676. 10.1182/blood-2002-08-2531 12406884

[B78] Medvinsky A. Dzierzak E. (1996). Definitive hematopoiesis is autonomously initiated by the AGM region. Cell 86, 897–906. 10.1016/S0092-8674(00)80165-8 8808625

[B79] Merad M. Manz M. G. Karsunky H. Wagers A. Peters W. Charo I.. (2002). Langerhans cells renew in the skin throughout life under steady-state conditions. Nat. Immunol. 3, 1135–1141. 10.1038/ni852 12415265 PMC4727838

[B80] Metchnikoff E. (1905). Immunity in Infective Diseases (Transl. from the French by Francis G. Binnie). Cambridge: Cambridge University Press.

[B81] Minematsu H. Shin M. J. Celil Aydemir A. B. Kim K. O. Nizami S. A. Chung G. J.. (2011). Nuclear presence of nuclear factor of activated T cells (NFAT) c3 and c4 is required for toll-like receptor-activated innate inflammatory response of monocytes/macrophages. Cell. Signal 23, 1785–1793. 10.1016/j.cellsig.2011.06.013 21726630 PMC3169434

[B82] Moore M. A. Metcalf D. (1970). Ontogeny of the haemopoietic system: yolk sac origin of *in vivo* and *in vitro* colony forming cells in the developing mouse embryo. Br. J. Haematol. 18, 279–296. 10.1111/j.1365-2141.1970.tb01443.x 5491581

[B83] Mouchemore K. A. Pixley F. J. (2012). CSF-1 signaling in macrophages: pleiotrophy through phosphotyrosine-based signaling pathways. Crit. Rev. Clin. Lab. Sci. 49, 49–61. 10.3109/10408363.2012.666845 22468857

[B84] Munro D. A. D. Hohenstein P. Davies J. A. (2017). Cycles of vascular plexus formation within the nephrogenic zone of the developing mouse kidney. Sci. Rep. 7:3273. 10.1038/s41598-017-03808-4 28607473 PMC5468301

[B85] Murray P. J. Allen J. E. Biswas S. K. Fisher E. A. Gilroy D. W. Goerdt S.. (2014). Macrophage activation and polarization: nomenclature and experimental guidelines. Immunity 41, 14–20. 10.1016/j.immuni.2014.06.008 25035950 PMC4123412

[B86] Muthukrishnan S. D. Ryzhova S. Karolaka M. Mukherjeeb E. Sims-Lucas S. Oxburgh L. (2017). A macrophage-based regenerative response to fetal kidney damage. Mech. Dev. 145:s50 10.1016/j.mod.2017.04.094

[B87] Nagata S. Hanayama R. Kawane K. (2010). Autoimmunity and the clearance of dead cells. Cell 140, 619–630. 10.1016/j.cell.2010.02.014 20211132

[B88] Palis J. Robertson S. Kennedy M. Wall C. Keller G. (1999). Development of erythroid and myeloid progenitors in the yolk sac and embryo proper of the mouse. Development 126, 5073–5084. 10529424 10.1242/dev.126.22.5073

[B89] Paolicelli R. C. Bolasco G. Pagani F. Maggi L. Scianni M. Panzanelli P.. (2011). Synaptic pruning by microglia is necessary for normal brain development. Science 333, 1456–1458. 10.1126/science.1202529 21778362

[B90] Penaloza C. Lin L. Lockshin R. A. Zakeri Z. (2006). Cell death in development: shaping the embryo. Histochem. Cell Biol. 126, 149–158. 10.1007/s00418-006-0214-1 16816938

[B91] Petrovic-Djergovic D. Popovic M. Chittiprol S. Cortado H. Ransom R. F. Partida-Sánchez S. (2015). CXCL10 induces the recruitment of monocyte-derived macrophages into kidney, which aggravate puromycin aminonucleoside nephrosis. Clin. Exp. Immunol. 180, 305–315. 10.1111/cei.12579 25561167 PMC4408165

[B92] Pollard J. W. (2009). Trophic macrophages in development and disease. Nat. Rev. Immunol. 9, 259–270. 10.1038/nri2528 19282852 PMC3648866

[B93] Rae F. Woods K. Sasmono T. Campanale N. Taylor D. Ovchinnikov D. A.. (2007). Characterisation and trophic functions of murine embryonic macrophages based upon the use of a Csf1r-EGFP transgene reporter. Dev. Biol. 308, 232–246. 10.1016/j.ydbio.2007.05.027 17597598

[B94] Ran S. Montgomery K. E. (2012). Macrophage-mediated lymphangiogenesis: the emerging role of macrophages as lymphatic endothelial progenitors. Cancers 4, 618–657. 10.3390/cancers4030618 22946011 PMC3430523

[B95] Rantakari P. Jäppinen N. Lokka E. Mokkala E. Gerke H. Peuhu E.. (2016). Fetal liver endothelium regulates the seeding of tissue-resident macrophages. Nature 538, 392–396. 10.1038/nature19814 27732581

[B96] Reinhoff W. F. (1922). Development and growth of the metanephros or permanent kidney in chick embryos. Proc. Natl. Acad. Sci. U.S.A. 33, 392–406. 3817290

[B97] Rogers N. M. Ferenbach D. A. Isenberg J. S. Thomson A. W. Hughes J. (2014). Dendritic cells and macrophages in the kidney: a spectrum of good and evil. Nat. Rev. Nephrol. 10, 625–643. 10.1038/nrneph.2014.170 25266210 PMC4922410

[B98] Rymo S. F. Gerhardt H. Wolfhagen Sand F. Lang R. Uv A. Betsholtz C. (2011). A two-way communication between microglial cells and angiogenic sprouts regulates angiogenesis in aortic ring cultures. PLoS ONE 6:e15846. 10.1371/journal.pone.0015846 21264342 PMC3018482

[B99] Sainio K. Suvanto P. Davies J. Wartiovaara J. Wartiovaara K. Saarma M.. (1997). Glial-cell-line-derived neurotrophic factor is required for bud initiation from ureteric epithelium. Development 124, 4077–4087. 9374404 10.1242/dev.124.20.4077

[B100] Sandberg M. L. Sutton S. E. Pletcher M. T. Wiltshire T. Tarantino L. M. Hogenesch J. B.. (2005). c-Myb and p300 regulate hematopoietic stem cell proliferation and differentiation. Dev. Cell 8, 153–166. 10.1016/j.devcel.2004.12.015 15691758

[B101] Sathi G. A. Farahat M. Hara E. S. Taketa H. Nagatsuka H. Kuboki T.. (2017). MCSF orchestrates branching morphogenesis in developing submandibular gland tissue. J. Cell Sci. 130, 1559–1569. 10.1242/jcs.196907 28348107

[B102] Sauter K. A. Pridans C. Sehgal A. Tsai Y. T. Bradford B. M. Raza S.. (2014). Pleiotropic effects of extended blockade of CSF1R signaling in adult mice. J. Leukoc. Biol. 96, 265–274. 10.1189/jlb.2A0114-006R 24652541 PMC4378363

[B103] Schnoor M. Cullen P. Lorkowski J. Stolle K. Robenek H. Troyer D.. (2008). Production of type VI collagen by human macrophages: a new dimension in macrophage functional Proc. Natl. Acad. Sci. U.S.A. 180, 5707–5719. 10.4049/jimmunol.180.8.5707 18390756

[B104] Schuchardt A. D'Agati V. Larsson-Blomberg L. Costantini F. Pachnis V. (1994). Defects in the kidney and enteric nervous system of mice lacking the tyrosine kinase receptor Ret. Nature 367, 380–383. 10.1038/367380a0 8114940

[B105] Schulz C. Gomez Perdiguero E. Chorro L. Szabo-Rogers H. Cagnard N. Kierdorf K.. (2012). A lineage of myeloid cells independent of Myb and hematopoietic stem cells. Science 336, 86–90. 10.1126/science.1219179 22442384

[B106] Seok S. J. Lee E. S. Kim G. T. Hyun M. Lee J. H. Chen S.. (2013). Blockade of CCL2/CCR2 signalling ameliorates diabetic nephropathy in db/db mice. Nephrol. Dial. Transplant. 28, 1700–1710. 10.1093/ndt/gfs555 23794669

[B107] Sheng J. Ruedl C. Karjalainen K. (2015). Most tissue-resident macrophages except microglia are derived from fetal hematopoietic stem cells. Immunity 43, 382–393. 10.1016/j.immuni.2015.07.016 26287683

[B108] Shi C. Pamer E. G. (2011). Monocyte recruitment during infection and inflammation. Nat. Rev. Immunol. 11, 762–774. 10.1038/nri3070 21984070 PMC3947780

[B109] Short K. M. Smyth I. M. (2016). The contribution of branching morphogenesis to kidney development and disease. Nat. Rev. Nephrol. 12, 754–767. 10.1038/nrneph.2016.157 27818506

[B110] Sieweke M. H. Allen J. E. (2013). Beyond stem cells: self-renewal of differentiated macrophages. Science 342:1242974. 10.1126/science.1242974 24264994

[B111] Stamatiades E. G. Tremblay M. E. Bohm M. Crozet L. Bisht K. Kao D.. (2016). Immune monitoring of trans-endothelial transport by kidney-resident macrophages. Cell 166, 991–1003. 10.1016/j.cell.2016.06.058 27477514 PMC4983224

[B112] Stewart K. Bouchard M. (2011). Kidney and urinary tract development: an apoptotic balancing act. Pediatr. Nephrol. 1419–1425. 10.1007/s00467-011-1788-y 21365192

[B113] Sumner R. Crawford A. Mucenski M. Frampton J. (2000). Initiation of adult myelopoiesis can occur in the absence of c-Myb whereas subsequent development is strictly dependent on the transcription factor. Oncogene 19, 3335–3342. 10.1038/sj.onc.1203660 10918590

[B114] Takahashi K. Yamamura F. Naito M. (1989). Differentiation, maturation, and proliferation of macrophages in the mouse yolk sac: a light-microscopic, enzyme-cytochemical, immunohistochemical, and ultrastructural study. J. Leukoc. Biol. 45, 87–96. 2536795 10.1002/jlb.45.2.87

[B115] Truman L. A. Ford C. A. Pasikowska M. Pound J. D. Wilkinson S. J. Dumitriu I. E.. (2008). CX3CL1/fractalkine is released from apoptotic lymphocytes to stimulate macrophage chemotaxis. Blood 112, 5026–5036. 10.1182/blood-2008-06-162404 18799722

[B116] Tsou C. L. Peters W. Si Y. Slaymaker S. Aslanian A. M. Weisberg S. P.. (2007). Critical roles for CCR2 and MCP-3 in monocyte mobilization from bone marrow and recruitment to inflammatory sites. J. Clin. Invest. 117, 902–909. 10.1172/JCI29919 17364026 PMC1810572

[B117] Vaage J. Lindblad W. J. (1990). Production of collagen type I by mouse peritoneal macrophages. J. Leukoc. Biol. 48, 274–280. 2202772 10.1002/jlb.48.3.274

[B118] van de Laar L. Saelens W. De Prijck S. Martens L. Scott C. L. Van Isterdael G.. (2016). Yolk sac macrophages, fetal liver, and adult monocytes can colonize an empty niche and develop into functional tissue-resident macrophages. Immunity 44, 755–768. 10.1016/j.immuni.2016.02.017 26992565

[B119] van Furth R. Cohn Z. A. (1968). The origin and kinetics of mononuclear phagocytes. J. Exp. Med. 128, 415–435. 10.1084/jem.128.3.415 5666958 PMC2138527

[B120] van Furth R. Cohn Z. A. Hirsch J. G. Humphrey J. H. Spector W. G. Langevoort H. L. (1972). The mononuclear phagocyte system: a new classification of macrophages, monocytes, and their precursor cells. Bull. World Health Organ. 46, 845–852. 4538544 PMC2480884

[B121] Varol C. Mildner A. Jung S. (2015). Macrophages: development and tissue specialization. Annu. Rev. Immunol. 33, 643–675. 10.1146/annurev-immunol-032414-112220 25861979

[B122] Yokomizo T. Dzierzak E. (2010). Three-dimensional cartography of hematopoietic clusters in the vasculature of whole mouse embryos. Development 137, 3651–3661. 10.1242/dev.051094 20876651 PMC2964097

[B123] Yona S. Gordon S. (2015). From the reticuloendothelial to mononuclear phagocyte system - the unaccounted years. Front. Immunol. 6:328. 10.3389/fimmu.2015.00328 26191061 PMC4486871

[B124] Zhan Y. Paolicelli R. C. Sforazzini F. Weinhard L. Bolasco G. Pagani F.. (2014). Deficient neuron-microglia signaling results in impaired functional brain connectivity and social behavior. Nat. Neurosci. 17, 400–406. 10.1038/nn.3641 24487234

